# Elvitegravir/Cobicistat/Emtricitabine/Tenofovir Alafenamide in the Treatment of HIV-Infected Patients: Experience with the First 100 Patients from Qatar

**DOI:** 10.1155/2020/1597839

**Published:** 2020-08-12

**Authors:** Hussam Al Soub, A. Latif M. Al-khal, Deema Alsoub, Waleed Awouda

**Affiliations:** Department of Medicine, Infectious Diseases Division, Hamad Medical Corporation, Doha, Qatar

## Abstract

**Background:**

To describe our experience with the use of Elvitegravir/Cobicistat/Emtricitabine/Tenofovir Alafenamide (EVG/COBI/FTC/TAF) in the treatment of HIV-infected patients in Qatar including both naïve and treatment experienced. We also report the reasons for switching to EVG/COBI/FTC/TAF in treatment-experienced patients, response to treatment, and tolerability.

**Method:**

Review of the medical records of the first 100 HIV-infected patients treated with EVG/COBI/FTC/TAF.

**Results:**

Among the 100 HIV-infected patients who were treated with EVG/COBI/FTC/TAF, 64 were Qatari and the rest were from other nationalities. 80 patients were males and 20 were females. 29 were treatment naïve, and 71 were treatment experienced. Among treatment-experienced patients, the most common reasons for switch to EVG/COBI/FTC/TAF were safety concerns, followed by regimen simplification and adverse drug reaction of the previous regimen (40%, 14%, and 13%, respectively). Treatment response to EVG/COBI/FTC/TAF leading to undetectable viral load in naïve patients was 69%, and in treatment-experienced patients, it was 83% with an overall response among all patients of 79%. Excluding those who left the country and whose data were not available, the response rate will be 86%. Tolerability was excellent with mild side effects and no discontinuation due to side effects.

**Conclusion:**

Experience with the use of EVG/COBI/FTC/TAF in 100 patients with HIV infection in Qatar was favourable both in treatment naïve patients and in those who were treatment experienced with an excellent tolerability.

## 1. Introduction

Antiretroviral therapy is indicated for all HIV-infected patients, including asymptomatic individuals, regardless of their immune status [1, 2]. Antiretroviral therapy (ART) reduces the progression to AIDS, opportunistic infections, hospitalizations, and death [3]. The goals of antiretroviral therapy are to reduce HIV-related morbidity and mortality and to prevent transmission of HIV to others. To achieve and sustain these goals, ART should result in maximal suppression of HIV RNA. Treatment of human immunodeficiency virus infection involves the use of combination antiretroviral therapy [2, 4]. More than 25 antiretroviral medications are available among six major classes. Existing studies have shown that antiretroviral drug adherence is essential to the maintenance of viral suppression and prevents hospitalizations, AIDS, and death [5, 6]. Among the various interventions to improve adherence to antiretroviral therapy was the introduction of fixed-dose combination or single-tablet regimens (STR). Once-daily, single-tablet regimens for the management of human immunodeficiency virus infection have become an integral part of initial antiretroviral therapy [7, 8]. They provide crucial advantages for the treatment of HIV. The most obvious advantage is the potential for improved adherence due to a lower pill burden.

Lower pill burdens have been associated with better virological suppression, and once-daily, single-tablet regimens can also improve patient satisfaction [9, 10]. A study of over 7,000 HIV-positive people found that those who take a single daily pill are less likely than those who take three or more daily pills to get sick enough to end up in the hospital [11]. In addition, fixed-dose combinations cut down on dosing errors. They also lower the likelihood that HIV will become resistant to the treatment. Currently, there are nine single-tablet regimens containing three agents to treat HIV infection. EVG/COBI/FTC/TAF which is a combination of Elvitegravir/Cobicistat/Emtricitabine/Tenofovir Alafenamide which was first approved by US the Food and Drug Administration (FDA) in 2015 as a single-tablet regimen for the treatment of HIV infection [12]. EVG/COBI/FTC/TAF was the first combination medication to use Tenofovir Alafenamide (TAF). TAF is less likely to cause kidney or bone problems than are associated with the use of Tenofovir Disoproxil Fumarate (TDF) [13, 14]. EVG/COBI/FTC/TAF was introduced in our formulary at Hamad Medical Corporation in 2016. EVG/COBI/FTC/TAF has been also introduced in other Gulf Cooperation Council (GCC) states nearly at the same time; however experience with its use has not been reported from any of these countries. In this study, we describe our experience in the use of EVG/COBI/FTC/TAF including the indications for change in those who were receiving other antiretroviral therapy, tolerance, safety, and outcome.

## 2. Materials and Methods

The study was conducted at the Hamad Medical Corporation (HMC), which is composed of eight hospitals with over 2300 beds distributed over the country and are the only governmental hospitals. We retrospectively studied all patients diagnosed with HIV infection in the period between 1984 and December 2018. HIV infection was diagnosed using the ELISA test as the screening test followed by western blot for confirmation. Patients were identified using our registry in the Compromised Host Clinic at the Communicable Diseases Center. Data collected included all of the following when available, age, sex, nationality, date of diagnosis, viral load at the time of starting EVG/COBI/FTC/TAF and 12–24 weeks later, CD + 4 cell count, complete blood count, renal and liver functions tests, antiretroviral treatment regimen, compliance and tolerability to the treatment, and outcome. It included also the date EVG/COBI/FTC/TA was started and the indication for changing to EVG/COBI/FTC/TAF in those receiving other treatments. The study was approved by the HMC Research Committee.

All HIV-infected patients in Qatar are managed and followed in the Communicable Diseases Center. Currently, we have 150 HIV-infected patients followed in the center. EVG/COBI/FTC/TAF is the most commonly used antiretroviral regimen at our center. Other commonly used combinations include Darunavir/Cobicistat plus Emtricitabine/Tenofovir Alafenamide, Dolutegravir plus Emtricitabine/Tenofovir Alafenamide, and the combination of Dolutegravir, Abacavir, and Lamivudine.

## 3. Results

Among the 100 patients who were treated with EVG/COBI/FTC/TAF at our center, 81 were males and 19 females. The mean age of the patients is 39.5 years. 64 were Qatari, and 36 were from other nationalities. 14 patients had their HIV diagnosis before the year 2000 and 86 after 2000. Twenty-nine patients were antiretroviral naïve upon starting EVG/COBI/FTC/TAF. Details of the patient's characteristics, previous treatment given, response EVG/COBI/FTC/TAF, and outcome are presented in Tables 1 and 2.

## 4. Discussion

In this study, we describe our experience with the first 100 patients that were treated with EVG/COBI/FTC/TAF at our center. Each EVG/COBI/FTC/TAF tablet consists of fixed doses of 150 mg Elvitegravir (EVG), 150 mg Cobicistat (COBI), 200 mg Emtricitabine (FTC), and 10 mg Tenofovir Alafenamide (TAF). EVG is an integrase inhibitor, which prevents viral replication by inhibiting the incorporation of viral DNA into host-cell DNA. COBI is a selective CYP3A inhibitor that is utilized in EVG/COBI/FTC/TAF as a pharmacokinetic booster for EVG and does not have any intrinsic activity against HIV. FTC is a nucleoside analogue reverse transcriptase inhibitor, while TAF is a nucleotide analogue reverse transcriptase inhibitor. TAF is a novel prodrug of tenofovir that undergoes intracellular conversion to tenofovir within target lymphoid tissue, thus limiting systemic tenofovir exposure, which may potentially improve long-term tolerability particularly related to renal impairment and reduced bone mineral density [13–15]. In phase III trials, EVG/COBI/FTC/TAF had noninferior efficacy to EVG/COBI/FTC/TDF in treatment-naive adults with regard to the rate of virological suppression at week 48 [16]. In treatment-experienced adults, switching to EVG/COBI/FTC/TAF was significantly more effective overall in maintaining virological suppression than ongoing treatment with tenofovir DF containing regimens. In particular, those who switched from Efavirenz/Emtricitabine/Tenofovir Df or Cobicistat- or Ritonavir-boosted Atazanavir plus Emtricitabine/Tenofovir DF had significantly improved rates of virological suppression at week 48, while those who switched from EVG/COBI/FTC/TDF (Stribild) maintained similarly high rates of virological suppression. EVG/COBI/FTC/TAF is generally well tolerated [17–19]. The overall incidence of drug-related adverse drug reactions was generally similar between TAF- and TDF-containing regimens; however, EVG/COBI/FTC/TAF is generally associated with significantly favourable effects on bone mineral density and renal parameters [17–19].

EVG/COBI/FTC/TAF has been approved for the treatment of HIV-1-infected adults and adolescents aged ≥12 years and weighing ≥35 kg by FDA in the USA in 2015 [12]. Current US guidelines now include EVG/COBI/FTC/TAF among the recommended regimens for ART-naive patients [20]. EVG/COBI/FTC/TAF has been added to our hospital formulary in 2016. We reviewed our experience with first 100 HIV-infected patients treated with EVG/COBI/FTC/TAF at our center. 80% of the patients were males, and 64% were Qatari. 29 patients were newly diagnosed to have HIV infection and were naïve for antiretroviral therapy, while the other 71 patients were treatment experienced. The main reasons for change of the regimen in treatment-experienced patients were safety concerns since all the previous regimens were containing Tenofovir Disoproxil Fumarate with special concerns regarding renal and bone side effects, followed by regimen simplification by changing from multitablet regimen to single-tablet regimen followed by adverse drug reaction from the previous regimen mostly related to (Efavirenz/Emtricitabine/Tenofovir DF) Atripla (40%, 14%, and 13%, respectively). The most common previous regimens were Atripla, EVG/COBI/FTC/TDF (Stribild), and Raltegravir/Emtricitabine/Tenofovir DF (32%, 18%, and 10%, respectively). Of the 29 treatment naïve patients, 20 (69%) responded to EVG/COBI/FTC/TAF with undetectable HIV viral load. 6 (21%) left the country soon after starting therapy so their response could not be assessed. Two patients failed treatment and were noncomplaint to their therapy. In the last patient, EVG/COBI/FTC/TAF was changed due to resistance to one of its components. In the 71 who were treatment experienced, 55 (77%) were having undetectable viral load at the time of switch to EVG/COBI/FTC/TAF. 50 (91%) of these patients continued to have undetectable viral load while 3 failed with detectable virus, all due to noncompliance to treatment, one patient died, and the last patient left the country before assessing his response. In those 15 patients with detectable viral load at the time of switch, 9 (60%) had undetectable virus, 4 (27%) continued to have detectable virus, 1 (7%) was changed due resistance to one component of EVG/COBI/FTC/TAF, and for another 1 (7%) patient, no data were available. By combining all patients, the response rate to EVG/COBI/FTC/TAF with undetectable viral load was 79%. The failure rate was 9% mostly due to nonadherence to treatment, and in 9%, the response could not be assessed due to leaving the country, death, or no data. In two patients (2%), the drug was changed because of resistance to one component of EVG/COBI/FTC/TAF. However, if only evaluable patients were included, the response rate will be 86%. There was also good response in CD + 4 cells rising from an average 633 cells at the start of EVG/COBI/FTC/TAF to 734 cells. The rise was observed in both treatment naïve (rising from an average of 640 to 728) and treatment-experienced (rising from average of 644 to 722 cells) patients. Tolerance to the drug was excellent with only three having mild side effects including abdominal pain, headache, and mild liver enzyme elevation, none of which lead to drug discontinuation. The effect on renal function was assessed only by measuring serum creatinine. All patients except 5 had a normal function at the start of EVG/COBI/FTC/TAF therapy. In patients with normal renal function at start of EVG/COBI/FTC/TAF, none had increase in serum creatinine. Among the 5 patients with abnormal function at start of treatment, 2 had slight elevation in serum creatinine, 2 had slight drop, and the fifth had no change. The two patients with increase in serum creatinine were also having diabetes mellitus. Effect on bone mineral density was not assessed in our patients.

In conclusion, our experience with the use of EVG/COBI/FTC/TAF in 100 patients with HIV infection in Qatar was positive both in patients who were treatment naïve and in those who were treatment experienced. The overall response rate was 79% and with 86% response in those patients who were evaluable. Overall tolerability and safety were excellent.

## Figures and Tables

**Table 1:** Baseline characteristics of HIV-infected patients.

Characteristics	Number
Sex	
Male	80
Female	20

Age	
Mean (years)	40
Range	14–80

Nationality	
Qatar	64
GCC	3
Pakistan	6
Philippine	5
Palestine	3
Others	19

Year of diagnosis	
<2000	14
>2000	86

Indication for switch to EVG/COBI/FTC/TAF	
New case	29
Side effects of previous regimen	13
Treatment failure	2
Drug interaction	1
Simplicity	14
Safety	40
Non‐compliance with previous regimen	1

Previous regimen	
Atripla	32
Darunavir/ritonavir plus Truvada	2
Atazanavir/ritonavir plus Truvada	3
Stribild	18
Kaletra plus Truvada	2
Raltegravir plus Truvada	10
Others	4

Year of starting EVG/COBI/FTC/TAF	
2016	49
2017	40
2018	11

Viral load at time of starting EVG/COBI/FTC/TAF	
Undetectable	55
Detectable	44
Range (51–836648 copies/ml)	
Not recorded	1

CD + 4 cell count at time of starting EVG/COBI/FTC/TAF	
Mean	633
Range	6–2286

**Table 2:** Response to EVG/COBI/FTC/TAF treatment and tolerability.

Effect on HIV viral load	Number

Treatment naïve	29
Undetectable	20
Left the country	6
Failure	2
Changed to another regimen due to resistance	1
Treatment experienced	71
Undetectable virus at the start (55 patients)	
Undetectable	50
Failure	3
Died	1
Left the country	1
Detectable virus at start (15 patients)	
Undetectable	9
Failure	4
Changed due to resistance	1
No data	1
No data at start (1)	
Failure	1

Effect on CD4	
Base line before starting EVG/COBI/FTC/TAF	
Mean	633
Range	(6–2286)
After starting EVG/COBI/FTC/TAF	
Mean	734
Range	(6–2335)

Adverse drug effects	
No side effects	87
Side effects	3
Headache	1
Abdominal pain	1
Elevated liver enzymes	1
Not evaluated	10

Effects on renal function	
Normal function	95
Abnormal	5
Improved	2
No change	1
Worsened	2

## Data Availability

The data used in this study can be made available from the corresponding author upon request.
